# Conservative and Surgical Treatment of Osteochondromas in Children, Particularly with or without Surgical Lengthening of the Ulna

**DOI:** 10.3390/jcm12134273

**Published:** 2023-06-26

**Authors:** Julie Mercier, Reto Bernasconi, Christina Steiger, Alexandre Kaempfen, Andreas H. Krieg

**Affiliations:** 1 Plastic, Reconstructive, Esthetic and Hand Surgery, Centre Hospitalier Universitaire Vaudevoise, Rue du Bugnon 46, 1011 Lausanne, Switzerland; julie.mercier@chuv.ch; 2 Anesthesiology Service, Ente Ospedaliero Cantonale, Via Tesserete 46, 6900 Lugano, Switzerland; reto.bernasconi89@gmail.com; 3 Paediatric Orthopaedics, Hopital Universitaire Geneve, Rue Gabrielle-Perret Gentil 4, 1205 Geneva, Switzerland; christina.steiger@hcuge.ch; 4 Childrens Orthopaedics, Universitäts Kinderspital Beider Basel, Spitalstrasse 33, 4056 Basel, Switzerland; andreas.krieg@ukbb.ch; 5 Plastic, Reconstructive, Esthetic and Hand Surgery, University Hospital Basel, Spitalstr. 21, 4031 Basel, Switzerland

**Keywords:** osteochondroma, forearm, lengthening, multiple hereditary exostosis, paediatric hand

## Abstract

Prevention of rotatory impairment and radial head dislocation in the forearm is an important aspect when treating children with osteochondromas. Various studies tried to determine the best treatment, describing different surgical techniques. No consensus has been reached yet. This retrospective study compares the treatment outcome of patients with osteochondroma of the radius and ulna after surgical or conservative treatment. Seventeen forearms treated over a period of 20 years were analysed. Outcome parameters were the prospectively collected clinical data and the radiological findings: “relative shortening” of ulna/radius, the “radial articular angle” (RAA) and the “carpal slip” (CS). Our study shows an improvement of the range of motion and cosmetic appearance of the forearm after an operative procedure, with or without bone lengthening. We observed an increase in wrist and elbow mobility with a decrease in pain scores and a confirmed high cosmetic satisfaction in almost 70% of the patients after bone lengthening and up to 85% after simple excision. For patients suffering from functional impairment or pain, an operative approach is beneficial. Multiple and repetitive osteochondroma excisions are recommended during growth to prevent deformity and rotatory motion restriction. Lengthening procedures require a careful indication.

## 1. Introduction

An osteochondroma is a benign cartilage-capped bone tumour and represents 30% of all benign bone tumours. The majority of osteochondromas occur in children as single lesions with equal distribution between genders, mostly in the femur, humerus, forearm, tibia, spine and hip, until the end of bone growth. They are usually located close to the epiphysis and proximally increase in size. Growth stops at skeletal maturity. 

Hereditary multiple osteochondromatosis of the skeleton (HMO), or hereditary multiple exostoses (HME), is a syndromic form of osteochondromas, characterised by the growth of multiple osteochondromas. It is a rare form of skeletal dysplasia with autosomal dominant inheritance. About 15% of all osteochondromas are found in the context of HMO. Around 1% of all of these benign osseous tumours degenerate into sarcomatous tumours, most commonly into chondrosarcomas [1,2]. 

Osteochondromas are often located in the forearm and in 30–60% of cases cause significant deformities [3]. The diagnosis of an osteochondroma is usually made by conventional X-ray. Typical radiological findings include relative shortening of the ulna, dislocation of the radial head, ulnar deviation of the hand, progressive ulnar translocation of the carpus, increased ulnar tilt of the distal radial epiphysis, as well as bowing of radius and/or ulna. 

Children presenting a full range of motion (ROM) without pain or deformity are treated conservatively. The conservative treatment consists of regular clinical and radiological follow ups and optimisation of ergonomics by the help of occupational therapists. Surgery is usually indicated to relieve pain, correct or prevent a functional deficit, or to improve cosmetic appearance. 

Most cases of solitary osteochondromas of the long bones are removed surgically by simple complete excision. However, if bone length is severely reduced in osteochondromas of the forearm, tumour excision with additional bone lengthening and correction of joint alignment may be considered. Vogt and Tretow recommend ulnar lengthening as early as possible to take advantage of the high remodelling potential of young children [4]. Bilen and Eralp suggest that osteotomy with gradual lengthening with an external fixator is the best method to treat these deformities [5], and Matsubara and Tshchiya describe largely improved function of the elbow and wrist articulation thanks to ulnar lengthening [6]. In contrast, other studies describe no benefit comparing forearm bone lengthening to simple tumour excision. A study by Akita and Murase demonstrated that ulnar lengthening only improves function and appearance of the forearm initially, but that deformities relapse, leading to no clinical and radiological improvement [7]. 

Therefore, we analysed our cohort of patients with osteochondromas in the forearm retrospectively in a long-term study. In our observation, conservative and surgical treatments consisting of a simple excision or an excision followed by bone lengthening were compared. 

## 2. Materials and Methods

The data of patients who were treated for osteochondromas in our orthopaedic clinic at the University Children’s Hospital were extracted from medical records and radiographs after approval by the local ethics committee (Ethical commission central and northern Switzerland Nr 2012/80). Clinical and radiological data were collected preoperatively, postoperatively during bone distraction, after fixator removal at standardised time intervals (6 weeks, 3 months, and 6 months) and late postoperatively (between 9 months and 21 years—mean 7.3 years).

Patients presenting a full or only slight reduction of ROM without pain or large deformity were closely observed.

Symptomatic patients always underwent either a simple excision of the exostoses, or excision and corrective procedures such as lengthening or corrective osteotomies of the radius or ulna, and rarely transport of the radius distally by lengthening of the ulna in cases of risk for or radial head dislocation. The surgical treatment was chosen depending first on ulna shortening and the impact of the deformity on the range of motion. Patients without ulna shortening were treated conservatively when the range of motion was not reduced. Otherwise, the patient underwent surgery. If the ROM was reduced, they underwent a prophylactic resection. Ulna shortening was divided into three categories: mild, moderate and severe. 

If a radial head was dislocated but clinically reducible and ulnar shortening was moderate or mild, a resection of the osteochondroma and lengthening of the ulna were performed. In cases of a non-reducible radial head dislocation, a radial head resection was performed. For analysis, the data were therefore split into three different treatment groups according to symptoms and clinical findings:

- (C) Conservative therapy,
- (E) Simple excision procedure,
- (L) Excision and lengthening.

All medical records and radiographs of the 15 patients (17 forearms) were furthermore retrospectively grouped depending on the location of the osteochondroma (proximal, central or distal), the type of treatment as explained above (conservative or operative with or without lengthening), the preoperative and postoperative mobility (pronation/supination, extension/flexion of the wrist, extension flexion at the elbow, ulnar/radial deviation of the hand), pain, cosmetic appearance, peri- or postoperative complications and recurrences of the osteochondroma. 

Anteroposterior radiographs of the entire forearm in full supination and pronation, completed by a lateral view of all four joints (elbow, wrist, distal and proximal radioulnar joints) are necessary to visualize the presence of osteochondromas and to define the deformities of the ulna and/or the radius. A quality check and calibration for standard radiographs ensured an accurate radiological interpretation of the images. All images with incorrect positioning or impossible measurements were excluded. 

**Figure 1:**
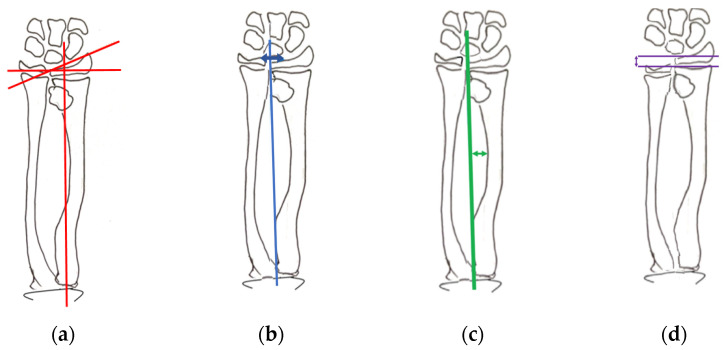
Radiological measurements: (**a**) radial articular angle, (**b**) carpal slip, (**c**) radial bowing and (**d**) relative ulnar shortening.

Several parameters, previously described by Shin and Jones [1], were measured in all selected radiographs:

- the radial articular angle (Figure 1a),
- the carpal slip (Figure 1b),
- the bowing of radius/ulna (Figure 1c),
- relative shortening of ulna/radius (Figure 1d),
- and the lengthening distance with the external fixator during the elongation period.

Variables (length gained, external fixation time (EFT), external fixation index (EFI) and distraction index (DI)) for patients undergoing bone lengthening were calculated according to a study by Matsubara and Tsuchiya. The DI was calculated by dividing the total duration of distraction by the length gained. The EFI was obtained by dividing the total duration of external fixation by the length gained [6]. 

Radial length can be measured as the distance from the centre of the proximal and distal radial margin from the lateral view. Ulnar length is also measured as the distance between the trochlea and the styloid of the ulna. Relative ulnar shortening was determined by subtracting ulnar length from radial length (Figure 1). 

The radial articular angle was measured between the line along the articular dorsal edge of the radius and a line starting on the radial edge of the epiphysis perpendicular to a line bisecting the proximal radial head (Figure 1). Normal values are between 15° and 30°.

Carpal slip is the percentage of the lunate surface in contact with the ulnar radial edge, limited by the axial line drawn from the ulnar edge of the radial head through the ulnar edge of the radial epiphysis (Figure 1). Normal values are >50%. 

Radial bowing (RB) is the greatest distance between the radial diaphysis and the axial line (Figure 1). Normal values are defined as <12 mm.

The active ROM was measured with a standard goniometer using established standards protocols to ensure reliable measurements. The following values were collected by a physician on every appointment: pronation/supination and extension/flexion of the wrist, extension/flexion of the elbow and ulnar/radial deviation of the hand. 

We used a Visual Analogue Scale (VAS) to measure pain intensity, 0 representing “no pain” and 10 “pain as bad as it could possibly be”. We also used a VAS scale from 0 to 10 to evaluate patient satisfaction pre- and postoperatively. 

All data is shown descriptively only in this small patient cohort with a non-normal data distribution. 

Complications were grouped according to the Clavien Dindo classification [8]. 

## 3. Results

### 3.1. Demographics

In the study cohort, 12 of 15 patients (13 forearms) were treated surgically, while three (four forearms) were treated conservatively. Simple excision of exostoses (nine ulnae, four radii) was the primary procedure in all patients (13 forearms) treated operatively. Simultaneous lengthening of the ulna was considered necessary in nine cases, corrective osteotomy of either the ulna or the radius was necessary in seven cases (six radii and one ulna) (Table 1). Only 3 of the 13 forearms were subject to more than one operation, all three due to complications. In addition, one of these forearms needed osteochondroma recurrence removal. All operations were performed before skeletal maturity. In our study cohort of 15 patients, 10 suffered from hereditary multiple osteochondromatosis. Four girls and 11 boys participated in the study. The mean age of the patients at the time of surgery was 9 years old. According to the Masada classification, there were three type I deformities, three type IIa, six type IIb and two type III. The remaining three forearms could not be assigned to a specific grade in the Masada classification because the lesions were located in the proximal ulna. This problem is frequently encountered and has recently been discussed by Farr et al. [9]. There was a total of nine radial head dislocations.

Four different types of fixators were used: three forearms were treated with the Wagner apparatus, one was treated with the Ilizarov appartus (Orthofix, Verona, Italy), two with the Hoffmann II external fixator System (Stryker Orthopaedics, Mahwah, NJ, USA) and the rest with the Monotube Yellow System (Howmedica Osteonics, Allendale, NJ, USA). 

Lengthening began 6 to 7 days after the surgery (except for one case, where it started after 24 days) and overall lasted 14 to 86 days (mean time 39 days).

### 3.2. Functional and Cosmetic Outcome

Patients treated conservatively neither perceived the osteochondroma nor the mild deformation as having a debilitating effect on their daily activities.

Analyses of medical records confirmed high subjective patient satisfaction after bone lengthening and after simple resection. An improvement in wrist and elbow movement and a decrease of pain was also encountered in 69% of the cases subjected to bone lengthening and in 85% of the cases undergoing simple osteochondroma resection. A decrease of elbow ROM (range 3° to 30°, mean 13.25°) was noticed in two forearms within the elongation group and a decrease of the wrist and elbow ROM, including a strength decrease, in one case within the simple resection group.

### 3.3. Radiographic Evaluation

#### 3.3.1. Relative Shortening

All eight patients (nine forearms) undergoing lengthening with an external fixator showed reduced relative ulna shortening at the final follow-up (Table 2). Relative shortening decreased during the lengthening period, had a maximum at the end of distraction and increased progressively thereafter until a final stable situation was reached after an average of 19.25 weeks. A stable situation was defined as a constant relative shortening value in two consecutive radiographs or after reaching skeletal maturity. Patients who did not undergo lengthening with an external fixator showed a steady increase in relative shortening.

#### 3.3.2. Radial Articular Angle

A reduction of the RAA was observed in the majority of cases undergoing lengthening of the ulna (mean RAA value of 39° before elongation to 35° at the final follow-up, with SDs of, respectively, of 5.3 and 6.4). In only one case, the RAA increased slightly after an initial decrease during the elongation period. In the remaining forearms, the RAA could not be measured due to incorrect positioning of the forearm on the X-ray. Patients with simple osteochondroma resection displayed an almost constant RAA. Only a brief diminution could be seen immediately after the operation, which returned quickly to the initial value (mean RAA value 34° at the beginning and 33° at the end, with a SD of respectively of 14.5 and 13.8) (Table 3).

Patients treated conservatively showed an RAA value increase during the entire follow-up period (Table 3).

#### 3.3.3. Carpal Slip

There was no difference in the carpal slip pattern between the two operatively treated groups. Both surgical interventions displayed a reduction of carpal slip. In the elongation group, four reductions and two improvements were observed (in the remaining forearms, carpal slip could not be measured due to incorrect positioning of the forearm in the radiograph). In the simple excision group, we could see a reduction of the carpal slip value in one case (in the remaining forearms, carpal slip could not be measured due to incorrect positioning of the forearm in the radiograph). In conservatively treated patients, carpal slip increased progressively (Table 3).

#### 3.3.4. Radial Bowing

Radial bowing in the elongation group showed similar values before surgery and at the final follow-up (difference <1% between initial and final radial bowing value) for one forearm. In four of nine cases, radial bowing increased. However, as with relative shortening, a decrease during the elongation period was noticed, followed by a steady increase after fixator removal. In the remaining forearms, radial bowing could not be measured due to incorrect positioning of the forearm in the X-ray (Table 3).

Patients undergoing a simple resection and those treated conservatively showed constant and physiological radial bowing in relation to radial length.

#### 3.3.5. Lengthening

Bone lengthening between 6 mm and 31 mm (mean 13.3 mm, SD 8.8 mm) was achieved in eight patients (nine forearms) (Table 2). After the elongation was stopped, a slight reduction in length was observed. Distraction length varied according to the type of external fixator used.

### 3.4. Complications of the Excision with Bone Lengthening Group

Seven complications occurred in six patients (six forearms) (Table 4). There were three pin tract infections (one during lengthening), one incongruence of the elbow joint due to radial head dislocation with night pain and pain on mobilization, one radial bowing due to ulna lengthening, one angulation in the osteotomy region, and one dislocation of the external fixator caused by a fall on the forearm resulting in ulna bowing. In four of the six patients, a re-operation was necessary (Clavien Dindo Classification Grade IIIb). In the first case, there was pain during lengthening associated with a light pin tract infection, which was successfully treated with antibiotics. Due to osteochondroma recurrence, there was an increase of relative ulna shortening that was treated with a second intralesional resection. Later on, radial head dislocation caused movement-related and night pain, requiring an ulna re-osteotomy and a radial head resection. In the second patient, an angulation at the osteotomy site was noticed, which was treated with an ulna re-osteotomy. One of the patients accidentally fell on the forearm, causing a dislocation of the external fixator leading to ulna bowing. A correction was obtained by ulna re-osteotomy and new placement of the external fixator. For the last patient, ulna lengthening caused a bowed radius movement inhibition that required a radial osteotomy. 

## 4. Discussion

Surgical versus conservative treatments of osteochondromas of the forearm in skeletally immature patients with regard to functional, cosmetic and radiological outcomes have been investigated in several studies. Preventing and reducing progression of deformity and functional impairment, particularly radial head dislocation, remain paramount goals [3]. However, no widely accepted therapeutic algorithm exists for this problem. A study by Klein et al. concludes that isolated osteochondroma resection should be reserved for symptomatic patients without significant forearm deformation and that patients suffering from forearm deformities should undergo surgery with radial corrective osteotomy [10]. Other studies, including ours, show that simple excision is effective in preventing progression of the deformity as measured in radiographs and improves the functional outcome [2].

Various studies on surgical corrective or reconstructive procedures comprising simple excision, acute or gradual lengthening of the ulna, corrective osteotomy of the radius and open reduction of the displaced radial head have been published. Many studies conclude that a simple and safe technique is preferable [10,11].

Excision of an osteochondroma, especially in young symptomatic patients, will improve the range of motion. However, by using this approach, surgeons have to accept the possibility of recurrences or new osteochondroma formations at the resection level, as excision of the osteochondroma does not alter the underlying disease [1]. Consequently, multiple operations during childhood may be required. In order to prevent this, less frequent operations with larger corrections, like bone lengthening procedures or osteotomies, could still be an alternative.

Bone lengthening is a delicate procedure requiring surgical experience. We observed a linear bone length increase with time during the elongation phase. However, once the external lengthening device is removed, a gradual decrease in length was encountered, which is in line with reports from other groups. Matsubara and Tsuchiya have therefore suggested performing overlengthening of the bone [6]. According to the authors, in order to achieve a correct final length, the unhealthy bone has to be elongated more than the approximate final length. In contrast, Vogt and Tretow have shown in their study that an overcorrection was inadvisable as it might cause an ulno-carpal impaction syndrome and, therefore, recommend accepting a certain degree of recurrence relying on high remodelling potential [4]. We did not notice an impaction syndrome in our patient cohort, but the ulna was lengthened to a neutral variance only.

Shin et al. also observed the progression of the disease in younger patients after simple excision and noticed no significant clinical improvement with ulnar lengthening. Therefore, alternatively to lengthening, he advised the combination of a Sauvé–Kapandji procedure with a simple excision of the osteochondroma as the best alternative to improve the function of the forearm, the stability of the wrist and the appearance of short ulnar forearms [1]. This was not an alternative for our patients as with ongoing growth, an arthrodesis and closure of the distal epiphysis did not seem appropriate.

Ip et al. pointed out the importance of calculating the optimal timing of surgery, which, in their opinion, was close to the time of calculated skeletal maturity [12]. Although some exostoses regress with skeletal maturity, they can still cause angular deformity and functional disability [13], which can be corrected after epiphyseal closure [10]. 

Peterson et al. recommend an early and aggressive surgical therapy [3]. Klein et al. concluded that children should be operated at the end of growth, because even though the deformity slowly returns during growth, the gained function after a surgical procedure remains good due to the adaptability of children [10].

In a study by K. Noonan et al., the function of the forearm in untreated patients with multiple hereditary osteochondromatosis [14] was examined at adulthood: osteochondromas can cause many complications such as altered osseous growth that leads to limb-length discrepancy, angular deformity, decreased range of motion and impaired function, but also eventually premature osteoarthritis. Not to mention the pain due to nerve, tendon or muscle irritation. The study also shows that adult patients subjectively adapted well to disability without having undergone surgery, and 87% of them were unrestricted in performing their chosen profession. However, the cosmetic outcome improved after a surgical procedure [14]. 

Our single-centre study was aimed at determining the effect of the different procedures used to treat osteochondromas in the forearm and to develop a treatment plan for future patients. 

The collected radiological and clinical data were retrospectively analysed. As previously shown by many authors, no improvement of the deformity has been detected in patients closely observed, which is the main reason why conservative and observational treatment is only recommended for minor deformities without clinical symptoms [4,5,6,7,15]. 

As in other studies, such as Matsubara et Tsuchiya, our study determined a significant improvement in function and cosmetic appearance of the forearm after an operative procedure, with or without bone lengthening [4,5,6,12,16,17]. 

We observed increased mobility of the wrist and elbow with decreased pain and a high cosmetic satisfaction. Our data confirm the results obtained by lshikawa et Kalo [18], who also found an increased functional and cosmetic outcome after simple tumour excision in patients with osteochondroma located in the distal part of the ulna or of the radius. 

In accordance with our radiological data, a few previous studies have shown a significant difference between patients treated with excision of exostoses and lengthening and those treated without bone-lengthening [4,5,12,16]. With regard to the relative shortening and the RAA, we found a remarkable improvement when excision of the exostosis was combined with radial/ulnar lengthening. Concerning carpal slip, we noticed an improvement in the excision and in the excision–elongation groups. Improvement of radial bowing was only observed during the elongation period. In contrast to other studies [4,5], no difference was noticed between the excision and the excision–elongation group for radial bowing and carpal slip at the end of the follow-up period. Similar results to ours were found in the studies of Akita and Murase, Shin and Jones, and Fogel and McElfresh [1,6,11]. Concerning functional differences, our clinical observations showed an increase in wrist and elbow mobility and a decrease of pain in the majority of the cases subjected to bone lengthening and also in those undergoing a simple osteochondroma resection.

All complications observed in this study occurred in patients undergoing bone lengthening, the most frequent of which being pin tract infections. 

Our retrospective study limitations are the small number of patients and the lack of radiographs with sufficiently standardised X-rays. The measures were also collected over a long period and some of them were therefore missing. A detailed statistical analysis of the data with a calculation of significance was not appropriate. The generalisation of our data is therefore limited.

General data collected longitudinally is difficult to judge, as our knowledge seems to increase gradually on these rare diseases. For example, a recent study by Farr et al. showed that using the classification of Jo [19] leads to a higher consensus among experts when grading forearms. It enabled classifying more cases and helped define the factors associated with radial head dislocation [8]. In the 1989 classification by Masada et al. [2], only the main osteochondromas are described, without considering additional osteochondromas. Farr et al., thus, suggest using the classification by Jo et al. [19] from 2017, which seems to be more precise. In our study, we were not able to reclassify the patients due to loss of the old primary X-rays.

## 5. Conclusions

In conclusion, no indication was found for an operative treatment in symptom-free patients whose ability to perform tasks of daily life are not affected and who presented no major deformities.

However, if a patient suffers from functional impairment or pain, we recommend, in line with other studies, an operative approach [20]. As both groups, with or without reconstructive bone lengthening, yield the same functional outcome regarding improved mobility of the wrist and elbow articulation, we recommend early simple excision due to the complications associated with lengthening. If necessary, this procedure has to be repeated multiple times during growth, but in series prevents the necessity for complicated lengthening and articular reconstructions of the forearm bones, which are cumbersome and prone to complications.

In our opinion, the main indication for bone lengthening is a recently dislocated radial head or a radial head at risk of dislocation. This would cause a functional impairment that can be improved or prevented by lengthening of the ulna with or without radius transportation in the distal direction.

## Figures and Tables

**Table 1:** Distribution of conservative vs. surgical therapy.

	15 Patients (17 Forearms)		
Conservative Therapy	3 Patients (4 Forearms)		
Surgery	12 patients (13 forearms)	Simple excision	4 patients (4 forearms)
		Excision with lengthening of the ulna	8 patients (9 forearms)
		Additional corrective osteotomy of radius or ulna	7 forearms

**Table 2:** Relative shortening and bone lengthening (mm) with mean, median and standard deviation values.

		Relative Shortening (mm)	Bone Lengthening (mm)
Excision with lengthening		10	6
		13	8
		2	11
		13	31
		15	6
		10	16
		14	15
	Mean	11.6	13.3
	Median	13	11
	SD	4.3	8.8

**Table 3:** Final measurements of the radial articular angle (in degrees), carpal slip (in %), radial bowing (in mm) in conservative, simple excision and lengthening groups at the last assessment with mean, median and standard deviation values (* not measurable due to non-standardised X-rays).

Therapy/Measurements		Radial Articular Angle (°)		Carpal Slip (%)		Radial Bowing (mm)	
Conservative		beginning	end	beginning	end	beginning	end
		27	37	49.1	50.9	13.5	15.6
		33	38	50.8	51.3	13.4	14.8
		40	44	34	37	13.7	14
		48	44	53.6	69.3	18.7	16.7
	Mean	37	41	46.9	52.1	14.9	15.3
	Median	36.5	41	50	51	14	15.5
	SD	8.9	4	8.8	13.2	2.6	1.2
Simple exostoses excision		preoperative	postoperative	preoperative	postoperative	preoperative	postoperative
		17	18	43.7	39.7	16.3	17.7
		*	32	*	26.1	*	12.2
		*	30	*	57.6	*	9.4
		53	51	*	*	24.7	25.8
		*	37	39.8	51.7	*	20.4
		45	36	49.9	41.2	11.2	14.1
		41	36	56.6	23	12.3	14.2
		38	36	54.7	32	15.2	18.9
	Mean	34	33	45.1	41.1	14	16.3
	Median	33.5	31	44	40	12	15
	SD	14.5	13.8	15.3	15.8	8.3	7.2
Excision with lengthening		38	35	38	50.5	8.1	8.7
		44	49	38.5	32.6	14	19.2
		39	35	*	30.3	12.1	*
		*	31	*	42.8	*	*
		*	32	*	51.7	*	*
	Mean	39	35	47.7	38	6.8	7
	Median	38.5	35.5	48	35	7	7
	SD	5.3	6.4	11.9	10.2	1.3	1.7

**Table 4:** Complications of the excision in the bone lengthening group.

Clavien/Dindo Classification	Complications	Number of Forearms
Grade I	Pin tract infections	3
Grade IIIb	Incongruence of the elbow joint due to radial head dislocation	1
Grade IIIb	Radial bowing due to ulna lengthening	1
Grade IIIb	Angulation in the osteotomy region	1
Grade IIIb	Dislocation of the external fixator caused by a fall on the forearm, resulting in ulna bowing	1

## Data Availability

Data available on request due to restrictions due to privacy in a small cohort and ethical limitations.
